# Dislocation of the Head of the Femur on the Dorsum Ilii, in a Child Aged Three Years and a Half

**Published:** 1849-01

**Authors:** 


*Dislocation of the Head of the Femur on the Dorsum Ilii, in a child aged three years and a half.*—Mr. Image, at the Provincial Medical Association, detailed the following rare and valuable case:

William Colley, aged three and a half years, of Ixworth, Suffolk, was admitted into the Suffolk Hospital, May 6th, 1847, with dislocation of the head of the femur on the dorsum ilii. The accident had occurred twelve days prior to his admission.

The accident appears to have been produced by his sister jumping upon his shoulders, from a step above that on which her brother was standing. When I first saw him the left great toe was resting on the instep of the opposite foot, and the knee crossing over the lower third of the right femur. The limb was an inch and three quarters shorter than the right. He was a remarkably fine healthy child. Soon after his admission reduction was attempted for a considerable time, but unsuccessfully, as we were not able to fix the knee-strap sufficiently tight to bear the long continued extension.

May 7th. Having obtained a proper sized knee-strap, and having nauseated our little patient with tartar emetic, and put him into a hot bath, we again applied the pullies, and after ten minutes succeeded in reducing the dislocation. The bone passed into its natural situation without the sligheSt jerk or sound. The limb now assumed exactly the same appearance as the other, the shortening being entirely removed. The thighs were kept tied together for a week, and on the 18th of May he walked out perfectly well.

I have ventured to lay the above case before the Society, in consequence of a charge having been urged against a neighbouring surgeon, of pretending to reduce a dislocation of the femur on the dorsum ilii, in a child only four years old, that child being a pauper and chargeable to the parish. It was agreed, and proved by authorities, that no such case was recorded, and therefore had not occurred, and that seven years' old was the earliest period at which this accident had taken place. On being referred to, I was rejoiced to have it in my power to lay before the proper authorities the above case, which was submitted to some leading surgeons in London, who admitted its perfect possibility, although it was considered to be a very rare occurrence.—*Ibid.*

